# Discrepancies in patients' medication lists from pharmacies in Sweden: an interview study before the implementation of the Swedish National Medication List

**DOI:** 10.1007/s11096-022-01480-x

**Published:** 2022-10-28

**Authors:** Tora Hammar, Leila Mzil, Birgit Eiermann

**Affiliations:** 1 eHealth Institute, Department of Medicine and Optometry, Faculty of Health and Life Sciences, Linnaeus University, Kalmar, Sweden; 2 Department of Pharmaceutical Biosciences, Division for Pharmacokinetics and Drug Therapy, Faculty of Pharmacy, Uppsala University, Uppsala, Sweden; 3 Department of Laboratory Medicine, Division of Clinical Pharmacology, Karolinska Institutet, Karolinska University Hospital Huddinge, Stockholm, Sweden; 4 Inera AB, Swedish Association of Local Authorities and Regions, Stockholm, Sweden

**Keywords:** Medication list, Electronic prescribing, Medication error, Medication reconciliation, Drug-related problems, Pharmacy

## Abstract

**Background:**

Discrepancies in medication lists are common and can contribute to drug-related problems. This study was performed before the implementation of the National Medication List in Sweden, an intervention expected to improve the accuracy of medication lists.

**Aim:**

The aim of the study was to examine the number and type of discrepancies in the medication list from pharmacies in Sweden. The secondary aim was to describe the information sources Swedish patients used as their medication lists and how confident they were with the information.

**Method:**

Structured interviews were conducted with patients at 13 community pharmacies in Sweden during the period October 5, 2020, to April 16, 2021. The printed medication list was reviewed together with the patient to identify any discrepancies and missing information.

**Results:**

A total of 327 patients were included in the study (response rate 51%). The printed medication list from pharmacies was the most common information source for patients to know which medications to use. Two thirds (*n  =  *215) of the patients had at least one discrepancy among their prescriptions and 32% (*n  =  *106) were missing at least one prescription medication. Among all prescriptions (*n  =  *2567) 10% (*n  =  *264) were non-current prescriptions, 9% (*n  =  *238) were duplicates and 3% (*n  =  *88) had the wrong dose. The proportion of prescriptions with discrepancies differed between drug-groups.

**Conclusion:**

The discrepancies described in this study can have serious consequences, and results provide a baseline for studies after the implementation of the National Medication List.

**Supplementary Information:**

The online version contains supplementary material available at 10.1007/s11096-022-01480-x.

## Impact statements

- Discrepancies in the medication list patients use to keep track of medications are common and could lead to drug-related problems.
- Two thirds of patients in the study had at least one discrepancy in their medication list from pharmacies.
- There is need to study if the National Medication List currently being implemented in Sweden will decrease the number of discrepancies as expected, or if additional interventions are needed.

## Introduction

Drug-related problems (DRPs) cause suffering for patients, are a common reason for hospital care, can be fatal and lead to substantial costs for society [1–4]. Many DRPs are possible to predict and avoid [5–7]. One reason for DRPs is that patients with many medications or frequent changes in treatment may have difficulties keeping track of the current treatment [8–10]. Providing patients with a correct medication list is important for safety and adherence. Unfortunately, errors and discrepancies in medication lists are a major problem internationally [11–17].

**Fig. 1:**
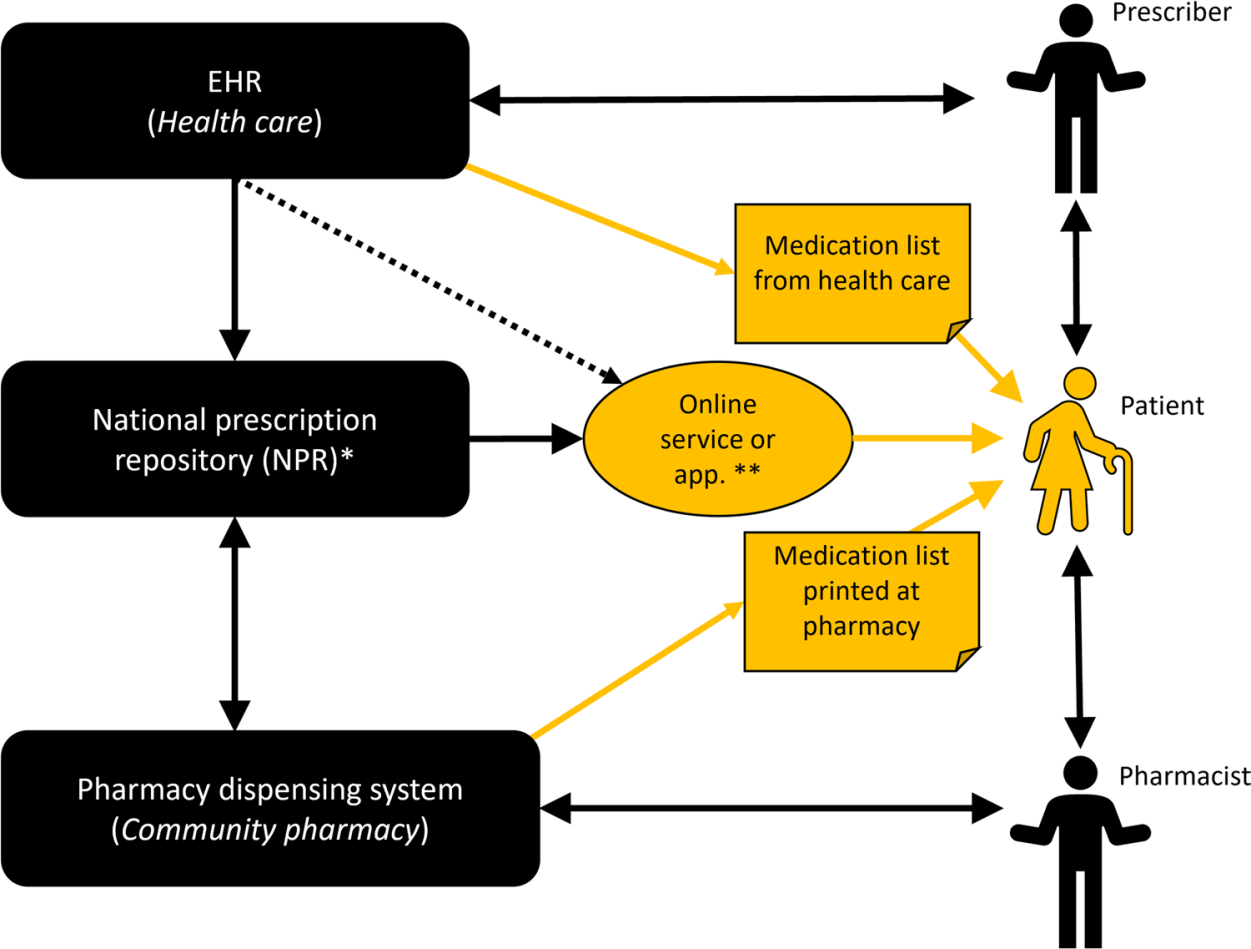
Overview of electronic prescribing in Sweden and the different sources of information that the patient can access, at the time of the study. Prescribers prescribe medications via a prescribing module in the Electronic Health Record (EHR) system. In Sweden many different EHRs are used, each having their own medication list. Due to insufficient interoperability between EHRs from different health care providers and between regions errors and missing information are common. When a new prescription is issued, the prescription is transferred to the national prescription repository (NPR), where it can be stored through the entire period of its validity (usually one year). Prescribers have not been allowed to view the patient’s prescriptions stored in the prescription repository due to legal reasons. From the NPR, the medication can be dispensed at any Swedish pharmacy. Pharmacists can, upon request from patients, view and dispense patients’ prescriptions via their dispensing system. Patients can view their electronic prescriptions via mobile applications or web pages using digital authentication. Patients can get medication lists printed in health care by for example their prescriber. Information in that list originates from the EHRs medication list. In addition, patients can get medication lists printed at pharmacies, the information then originates from the NPR (*which is the medication list in focus in this study and often differ from the EHR medication list*). Dispensed medication packages have labels printed at pharmacies with dosage instructions from the NPR. *The NPR has been replaced with the National medication list since May 2021 (after the present study was performed), and prescribers are now allowed to view this information. **The information available to patients via online services and applications can originate from either the NPR or the EHR. The patient electronic health record online (called 1177 journalen) includes information from the NPR and the EHR as two separate lists, the medication lists in the online service “Läkemedelskollen” from the Swedish E-Health Agency and services from pharmacy chains all originates from the NPR

In Sweden patients can get medication lists printed from health care (i.e. their physicians, from pharmacies or access online). However, these lists often differ in content and accuracy. More than 99% of all prescriptions in Sweden are electronic and stored in a national register (Fig. 1) called the National Prescription Repository (NPR) [18, 19]. Prescribers have medication lists for their patients in their electronic health records (EHR). Due to insufficient interoperability between EHRs from different health care providers and between regions errors and missing information are common [14, 18, 20]. The medication lists from health care should be the primary source of information for patients. In reality, patients often use the medication lists from pharmacies instead, which might be a risk to patient safety [9]. Additionally, patients can access their medication list digitally through several different services.

Previous Swedish studies have found that more than 80% of patients had at least one discrepancy in their medication list from healthcare and pharmacies and that overall congruence between the lists was only 55% [13, 20]. Studies from other countries also show a high prevalence of discrepancies in medication lists [12, 21].

One approach to reduce the number of discrepancies is by using a shared medication list [22]. Several countries have implemented, or are about to, digital solutions for a shared medication list [11, 23, 24]. A recent study from Norway found that an electronically shared medication list greatly decreased discrepancies between primary care, home care and pharmacy medication lists. [25].

In an attempt to improve the situation in Sweden and increase patient safety the National Medication List is going to be implemented in health care and pharmacies. A new law (*Lag (2018:1212) om nationell läkemedelslista*) was put in force May 1, 2021 [26]. The National Medication List contains in its first version all prescriptions but no information about drugs administered in hospitals and no OTC drugs. The National Medication List is not yet integrated in EHRs in Sweden. One of the presumed benefits is decreasing the number of discrepancies. The present study was performed before the new law was in place, focusing on the information in the register (NPR) that forms the basis for the new National Medication List [26]. To be able to study the effects of the national intervention over time there was a need for more recent data on the discrepancies in the list.

### Aim

The aim of the study was to examine the number and type of discrepancies in the medication list from pharmacies in Sweden. The secondary aim was to describe the information sources Swedish patients use as their medication lists and how confident they are with this information.

### Ethics approval

This study has been approved by the Swedish Ethical Review Authority as a part of a larger project about effects from the implementation of the National Medication List (Dnr 2019-06553 with decision 2020-03-09 and Dnr 2020-04017 -*amendment* with decision 2020-08-11). Informed consent was collected from all patients in the study before participation.

## Method

Structured interviews were conducted with patients at Swedish community pharmacies to examine the number and type of discrepancies in their printed list of prescriptions, and to receive information about sources they use to know which their current medications are and how confident they are with the information. The study was designed to provide a baseline before the national implementation of a shared medication list, to enable future comparison to examine if the implementation affects the number and type of discrepancies in the printed medication list from pharmacies.

### Study population

The study was conducted at 13 community pharmacies in Sweden by six pharmacy students during the period October 5, 2020, to April 16, 2021. The pharmacies were strategically selected, belonged to different pharmacy chains, spread geographically across Sweden in seven cities of varying population size, and included pharmacies of different sizes. Data was collected at each pharmacy for a couple of days up to two weeks, at different time points during the day.

The inclusion criteria for patients in the study were: (1) age 18 years or older, (2) collecting medications for themselves, (3) three or more prescriptions in their medication list, (4) speaking Swedish, (5) having ordinary prescriptions, not multi-dose drug dispensing.

Patients visiting any of the pharmacies to collect prescription medications during data collection who met the inclusion criteria were asked to participate in the study by the dispensing pharmacist who also provided short information about the study. For patients who agreed to participate, the medication list from the NPR was printed and a consent form was signed before the interview was performed in a secluded place of the pharmacy. To calculate response rate, pharmacists kept track of patients meeting inclusion criteria that declined to participate.

### Data collection

**Fig. 2:**
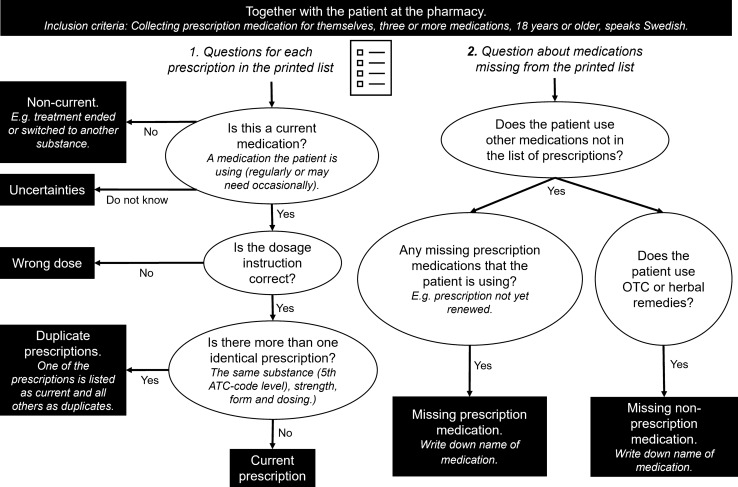
Flow-chart for review of printed medication list from pharmacies. Each prescription could only be classified as one of the categories, either current prescription or one of the discrepancies. (OTC = over the counter medications; drugs which can be bought without any prescription)

The structured interview followed an interview guide (Supplementary electronic material) including four parts:

1. Background information: age and gender
2. Three questions about which medication lists and information sources they had used the previous year to know which medications they should take and in what dose. Multiple choice question with nine alternatives and the possibility to answer outside of the given alternatives.
3. Three questions about how confident they feel about which medicines they should use, how they should use them and why. Answers were given on a five-point Likert scale where 1 represented ”No, not at all” and 5 ”Yes, completely”.
4. Review of the printed medication list from pharmacies to identify any discrepancies, any missing medications and identification of possible use of OTC-drugs (Fig. 2).

The filled in interview guides were saved together with one copy of the printed list of prescriptions with notes regarding discrepancies according to the flow-chart in Fig. 2. Names and personal ID were removed from these documents.

### Analysis

Data was analyzed using IBM SPSS Statistics 27 and Excel 365. Due to non-normality among the dependent variables non-parametric tests were applied. For continuous variables the Mann–Whitney u-test was used and for proportions the chi2-test. Binary variables were used for gender (male and female) and age (< 70 and ≥ 70 years). Differences in discrepancies between different drug groups was analyzed using the Anatomical Therapeutic Chemical (ATC) code classification system [27]. In the ATC classification the active substances classified in groups at five different levels. In the present study we used the 2^nd^ level where drugs are divided into pharmacological or therapeutic subgroups.

## Results

A total of 642 patients met the inclusion criteria and were asked to participate and 327 of them agreed, giving a response rate of 51%. The included patients (*n = *327) had a mean age of 68 years (ranging from 20 to 94 years, STD 14.9), 61.5% were women and 38.5% were men. Participants had a total of 2567 prescriptions in the printed medication lists from pharmacies (Table 1).

**Table 1:** Description of the study population (*n = *327)

Category	N (%)
*Gender*	
Female	201 (61.5)
Male	126 (38.5)
*Age*	
< 30	16 (4.9)
30–39	4 (1.2)
40–49	14 (4.3)
50–59	36 (11.0)
60–69	71 (21.7)
70–79	126 (38.5)
80–89	54 (16.5)
> = 90	6 (1.8)
*Number of prescriptions*	
Less than 5	72 (22.0)
5–10	184 (56.3)
More than 10	71 (21.7)

### Discrepancies in medication lists from pharmacies

Of the 327 patients, 65.7% had one or more discrepancies (non-current, duplicates and/or prescriptions with wrong dose), among the prescriptions in their printed medication list from the pharmacy (Table 2). Thus, the rest (34.3%) did not have any discrepancies in their list. The average number of discrepancies among all patients were 1.83 (std 2.315). The number of discrepancies per patient ranged from 0 to 16, where 44% of patients had 2 or more discrepancies in their list. There was no difference in number of discrepancies related to age (*p = *0.263) or gender (*p = *0.153). In addition, 106 patients (32.4%) were missing at least one prescription medication in their printed list from pharmacies. If missing prescriptions are calculated together with the other discrepancies, 77.7% (*n = *254) of all patients have at least one discrepancy (Table 2).

**Table 2:** Prevalence of different types of discrepancies

Type	Prescriptions *n* (%)	Patients *n* (%)
Non-current prescriptions	264 (10.3)	129 (39.4)
Duplicate prescriptions	238 (9.3)	110 (33.6)
Wrong dose	88 (3.4)	62 (19.0)
Uncertainties	5 (0.2)	5 (1.5)
Any discrepancy (excluding missing prescriptions)	595 (23.2)	215 (65.7)
*Any discrepancy (including missing prescriptions)*	752 (27.6**)	*254 (77.7)*
*Missing prescriptions*	157 (5.8**)	*106 (32.4)*
Total*	2567* (100)	327

^*^Total number of prescriptions does not include missing prescriptions, only prescriptions present in the printed medication list from pharmacies

^**^proportions (%) calculated with the denominator 2724 (i.e. the total number of prescriptions in list and the total number of missing prescriptions)

Among all prescriptions (*n = *2567), 23% (*n = *595) of the prescriptions had some form of discrepancy where 10.3% were non-current prescriptions, 9.3% were duplicates and 3.4% of the prescriptions had the wrong dose according to the patient (Table 2).

### Discrepancies among different groups of medications

The 20 most frequent prescribed drugs on the 2nd ATC code level cover 80% of all prescriptions among the participants, 83.0% of all prescriptions with wrong dose, 85.3% of all duplicate prescriptions but only 67.0 of all non-current prescriptions (Table 3). *Drugs acting on the renin-angiotensin system* (ATC C09) were the most common group of medication (*n = *189) representing 7.4% of all prescriptions in the study.

**Table 3:** The table contains the 20 most common drug groups, on the 2nd ATC code level, among all prescriptions in the study

ATC code and name of drug group	All prescriptions	Non-current prescriptions	Duplicate prescriptions	Prescriptions with wrong dose
	*n* (% of all prescriptions)	*n* (% within ATC group)	*n* (% within ATC group)	*n* (% within ATC group)
C09—Agents acting on renin-angiotensin syst	189 (7.4)	7 (3.7)	21 (11.1)	4 (2.1)
N02—Analgesics	172 (6.7)	20 (11.6)	17 (9.9)	12 (7.0)
C10—Lipid modifying agents	170 (6.6)	9 (5.3)	13 (7.6)	1 (0.6)
A10—Alimentary tract and metabolism	160 (6.2)	4 (2.5)	21 (13.1)	11 (6.9)
C07—Beta blocking agents	132 (5.1)	5 (3.8)	12 (9.1)	4 (3.0)
B01 – Anti-thrombotic agents	126 (4.9)	1 (0.8)	9 (7.1)	0 (0.0)
C08—Calcium channel blockers	113 (4.4)	12 (10.6)	14 (12.4)	1 (0.9)
R03—Drugs for obstructive airway diseases	112 (4.4)	13 (11.6)	7 (6.3)	4 (3.6)
S01—Ophtalmologicals	106 (4.1)	24 (22.6)	15 (14.2)	1 (0.9)
A02—Drugs for acid related disorders	97 (3.8)	5 (5.2)	8 (8.2)	5 (5.2)
N05—Psycholeptics	93 (3.6)	17 (18.3)	9 (9.7)	4 (4.3)
B03—Antianemic preparations	90 (3.5)	3 (3.3)	10 (11.1)	2 (2.2)
N06—Psychoanaleptics	81 (3.2)	7 (8.6)	7 (8.6)	8 (9.9)
C03—Diuretics	78 (3.0)	3 (3.8)	9 (11.5)	2 (2.6)
R06 – Anti-histamines for systemic use	63 (2.5)	6 (9.5)	6 (9.5)	2 (3.2)
H03—Thyroid therapy	60 (2.3)	1 (1.7)	7 (11.7)	4 (6.7)
G04—Urologicals	59 (2.3)	14 (23.7)	2 (3.4)	0 (0.0)
A06—Drugs for constipation	57 (2.2)	7 (12.3)	8 (14.0)	4 (7.0)
G03—Sex hormones, modulators of the genital system	52 (2.0)	5 (9.6)	5 (9.6)	2 (3.8)
M01 – Anti-inflammatory and antirheumatic	45 (1.8)	14 (31.1)	3 (6.7)	2 (4.4)
Total number of prescriptions among the 20 ATC (% of all prescriptions within category)	2055 (80.0)	177 (67.0)	203 (85.3)	73 (83.0)
Total in study (% of all prescriptions)	2567 (100)	264 (10.3)	238 (9.3)	88 (3.4)

For each category of discrepancy (wrong dose, duplicates, and non-current drugs) the number of prescriptions with the discrepancy is given, together with percentage within the ATC group

Among the 20 most common drug groups the highest proportion of prescriptions with wrong dose was found in the drug group *Psychoanaleptics* (ATC N06) where 9.9% of all prescriptions had incorrect dosing instructions. The highest proportion of duplicate prescriptions were found among *Drugs for constipation* (ATC A06) and *Ophtalmologicals* (ATC S01) with 14.0 and 14.2% being duplicates, respectively. Non-current prescriptions occurred mainly within *Antiinflammotory and antirheumatic products* (ATC M01) with almost one third of all prescriptions (31.1%), followed by *Urologicals* (ATC G04) with almost one quarter (23.7%), being non-current.

### Use of non-prescription (OTC) medications

The printed medication list includes only prescription medications and no OTC drugs. Among the patients in this study 55% (*n = *180) reported that they use OTC drugs, and 100 of them specified the OTC drugs they take. The most common drugs used are analgesics (N02) which are taken by 43% of the 100 patients (most common paracetamol) followed by various vitamins (A11) which are taken by 31% and antiinflammatory and antirheumatic products (M01) taken by 20% of the patients (most common ibuprofen). In total drugs from 19 various ATC codes on 2nd level are used.

### Information sources used by patients

**Fig. 3:**
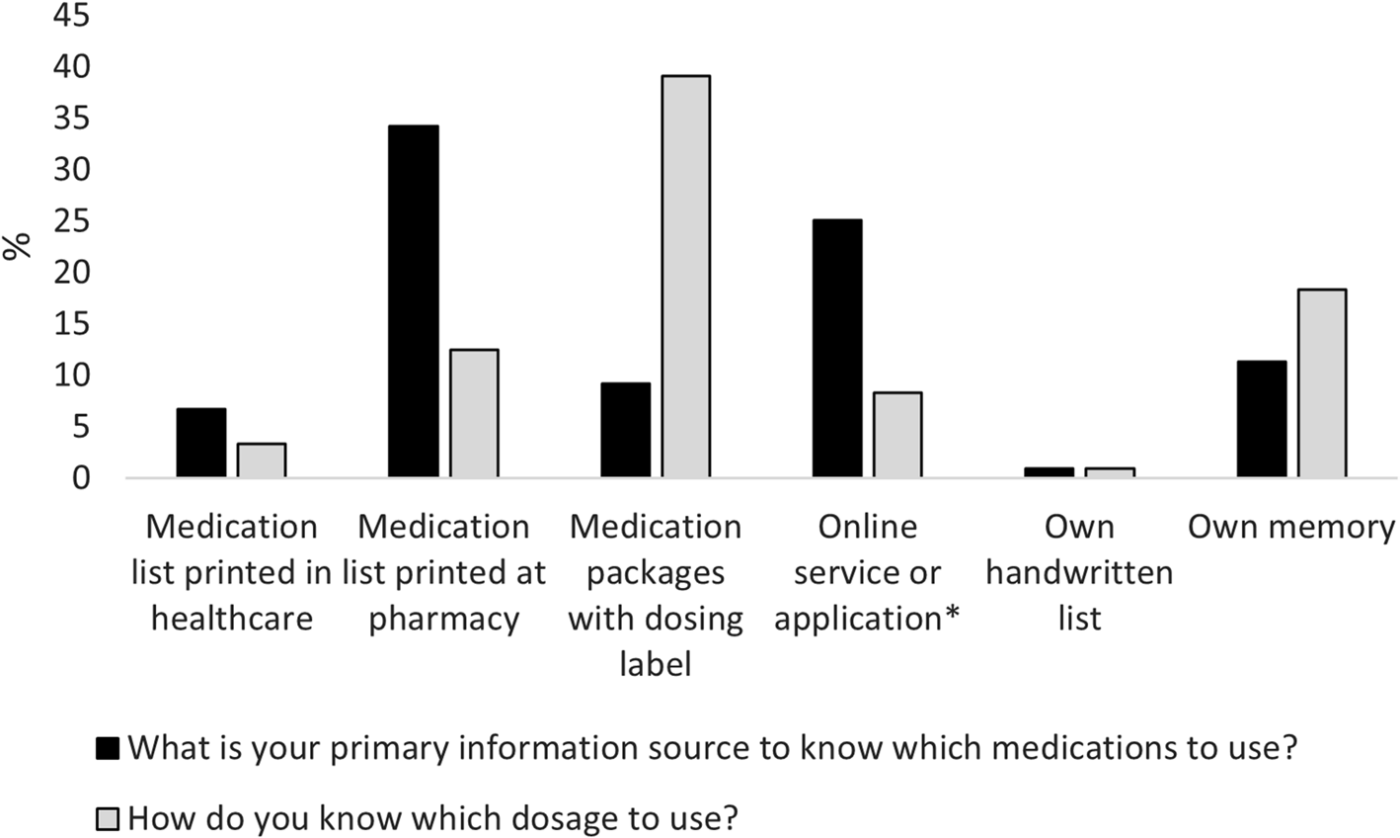
Information sources used by patients to know which medications to use and in which dosage. Presented as proportion of all patients in the study. Several answers were possible for each respondent. The alternative “Online service and application” included the patient electronic health record “1177 Journalen”, the online service “Läkemedelskollen” from the Swedish E-Health Agency and services from pharmacy chains

The information source most patients reported they had received or accessed in the past year to get an overview of their medications was the printed medication list from pharmacies (Fig. 3). This list was also the most frequently reported as the primary source for knowing which medications they should use. The most common information source to know which dosage to use was the medication package with the label containing dosage instructions from the prescription (also printed at the pharmacy). Among other information sources mentioned, oral information from health care professionals was used primarily.

### Confidence with information about medications

More than three quarters of patients felt confident with their medication related information, answering “Yes, completely” to the following three questions (i.e. a five on the five-point Likert scale); “Do you feel confident about which medicines you should take?” (79%, *n = *257), “Do you feel confident about how to take all your medicines (87%, *n = *284), and “Do you feel confident about why you should take your medicines?” (85%, *n = *278).

## Discussion

The study shows that about two thirds of patients had at least one discrepancy among the prescriptions in their printed medication list from pharmacies, including non-current prescriptions, duplicates and prescriptions with the wrong dose. In addition, one third of patients were missing at least one prescriptions medication in the list and more than half reported that they use OTC drugs. Discrepancies differed between the different groups of medications. Wrong dose was most common in prescriptions within the ATC code group *Psychoanaleptics*, duplicate prescriptions were most commonly found among *Ophtalmologicals* and non-current prescriptions occurred mainly within *Antiinflammotory and antirheumatic products*.

The printed medication list from pharmacies was the most common information source for patients to know which medications to use. Still, most patients felt confident about which medicines to use, how to use them and why they should take their medications.

### Strengths and weaknesses

This study used patients’ own knowledge to examine discrepancies, missing prescriptions and OTC drugs which is not necessarily the truth. To draw stronger conclusions about OTC drug use of other methods with more structured ways of getting this information is probably necessary. Our method differs slightly from previous Swedish studies where patients were informed about the study in advance and were asked to spontaneously report their current medications before looking at the list [13, 20]. This might explain why we identified fewer missing prescriptions in this study.

The present study was performed during the Covid pandemic which led to some challenges in data collection and recruiting of patients. It has been shown previously that including patients in research when they collect medications at pharmacies can lead to the elderly being underrepresented [28]. During the pandemic even fewer of the old patients visited pharmacies themselves due to risk of infection. Thus, our results may not be generalizable for the old and frail patients. Studies after the implementation of the National medication list may need to be adjusted with regard to the age of the study population to enable comparison.

### Interpretation and comparison with previous research

Similar to other studies discrepancies were common. In the present study 66% had at least one discrepancy among the prescriptions in their printed list, and if missing prescriptions are calculated together with the other discrepancies, 78% of all patients have at least one discrepancy. These numbers are a bit lower than a previous study in Sweden where 87% of patients had at least one discrepancy in their medication list from the pharmacy, and 83% had at least one discrepancy in the medication list from EHR [20]. The findings are similar to results from Denmark where 75% of patients admitted to hospitals had at least one discrepancy in their medication list from electronic prescribing systems [12]. In both the previous Swedish and Danish study non-current prescriptions, wrong dose, duplicates, and missing prescriptions are common, although the proportion of patients with these discrepancies differ between studies [12, 20].

The explanation to the many discrepancies lies in the integration of the NPR (or rather the lack of it) with EHRs in health care and the fact that prescribers have not been allowed to access this list. When the study was performed the communication between the EHR and the register was one-way, i.e. new prescriptions were transferred from EHR to the NPR. Therefore, if a prescriber for example decided to change the dose of a medication in the EHR but did not send a new prescription this change would not be visible in the NPR. Furthermore, if a prescriber does send a new prescription when changes are made, the old prescription is still included in the NPR if not actively removed by the prescriber. Thus, the printed list of prescriptions that patients receive at the pharmacy can include prescriptions for medications that the patients should no longer use (non-current), prescriptions with incorrect dosing instructions and several prescriptions for the same medication if a new prescription is sent before the old one is ended. In addition, this list only includes valid prescriptions and may be missing outdated prescriptions for medications that the patients should be using. In Sweden, prescriptions are usually valid for one year or until all the medication is dispensed. Prescriptions must be renewed after that, otherwise they will not be included in the printed list from pharmacies.

Only medications that have been prescribed are included in the medication list from pharmacies. Non-prescription medications, like OTC-drugs, are never included in this list as well as drugs administered in hospitals. Our results show that many patients take OTC drugs, and analgesics are the most common ones. These are highly potent drugs which are also commonly prescribed and can cause DRPs such as side effects and drug interactions [29, 30]. OTC drugs will not be included in the National shared medication list in the beginning, but this should be considered in the future along with medications administered in the hospitals.

The results are in line with previous studies showing that this printed list from pharmacies are used most frequently by patients despite not being intended or designed to be used like that [9]. On the other hand, the medication lists from health care EHR are often incomplete due to the fact that patients might go to several physicians [18]. This will hopefully change and improve with the introduction of the National Medication List in Sweden. The present study shows that the most common information source used by patients to keep track of what dose to take was the dosing instructions printed on the label for patients’ medication packages. This information also originates from the NPR, meaning that the incorrect doses found in this study also apply to this. Discrepancies can also increase the risk of dispensing medications that the patient should no longer be taking or including incorrect dosing instructions [31]. Despite all the problems more than three quarters of the patients are feeling secure in what drug they should use, how they should use them and why they should use them.

### Further research

The expectations on the National Medication List currently being implemented in Sweden are high and include the goal that it should lead to fewer discrepancies in the medication lists available in health care, at pharmacies and for patients [32]. Using the method and results from the present study effects should be studied over time. Future studies should also use other quantitative and qualitative methods to study the effects of the National Medication List in Sweden, as well as the similar solutions in other countries.

## Conclusion

Among patients collecting prescription medication at Swedish pharmacies, about two thirds had at least one discrepancy among the prescriptions in their printed medication list from pharmacies. The most common discrepancy was non-current prescriptions in the list, followed by duplicates and prescriptions with the wrong dose. Discrepancies differed between the different groups of medications. In addition, one third of patients were missing at least one prescription’s medication in the list. The printed medication list from pharmacies was the most common information source for patients to know which medications to use. Despite the many errors in the list, most patients felt confident about which medicines to use, how to use them and why they should take their medications. This study was performed before the implementation of the National Medication List in Sweden and was designed to provide a baseline to enable future studies to examine if the number of discrepancies decrease after the national implementation.

## Supplementary Information

Below is the link to the electronic supplementary material.

Supplementary file1 (DOCX 28 kb)
