# Supplementary material for: Discrepancies in patients' medication lists from pharmacies in Sweden: an interview study before the implementation of the Swedish National Medication List
Source: Int J Clin Pharm. 2022 Oct 28;45(1):88–96. doi: 10.1007/s11096-022-01480-x (PMC9938824; doi:10.1007/s11096-022-01480-x)
Supplement: Supplementary file 1 — Supplementary file1 (DOCX 28 kb) [file 11096_2022_1480_MOESM1_ESM.docx]

**Interview guide**

Date:

Pharmacy:

1. **Gender**   male  female  other
2. **Age** ___________
3. **Which medication lists have you received or used the last year?**

Printed medication list from health care

Printed medication list from pharmacies (*Mina sparade recept*)

Medication packages with dosing label

Patient electronic health record (*1177 Journalen*)

My saved prescriptions through the digital service from E-Health Agency (*Läkemedelskollen*)

Pharmacy chains websites or mobile apps

Historical information about dispensed prescriptions (*Läkemedelsförteckningen*)

Own handwritten list

Own memory

Other: ________________________

1. **Which information sources do you use mainly to know, which drugs you should take?** _______________________________________________________________________
2. **What do you do to know which dose you should take of your drugs?** _______________________________________________________________________
3. ***What does the patient feel about their drug use on a scale from 1-5 where 1 means ”No, not at all” and 5 ”Yes, completely”:***

Do you feel confident about which medicines you should take?  1  2  3  4  5  Not relevant Do you feel confident about how to take all your medicines?  1  2  3  4  5  Not relevant

Do you feel confident about why you should take your medicines?  1  2  3  4  5  Not relevant

1. **Review of medication list (see flow chart, figure 1)**
2. Check medication list together with participant and check in the list for each prescription if it is current, non-current, wrong dose, duplicate or uncertainties.

Mark **c= current**, **n= non-current, w= wrong dosage**, **d= duplicate**, **u= uncertainty**

1. **Are any prescribed drugs you use missing on the medication list?**  Yes  No

If ”yes” which ones: ________________________________________________________________

1. **Do you use any OTC drugs (over the counter = non-prescription drugs)?**  Yes  No

If ”yes” which ones: ________________________________________________________________

1. **Do you have any comments or wishes regarding the printed medication list from pharmacies?**

_______________________________________________________________________

_______________________________________________________________________
